# Accelerated mineralization on nanofibers via non-thermal atmospheric plasma assisted glutamic acid templated peptide conjugation

**DOI:** 10.1093/rb/rbz014

**Published:** 2019-04-22

**Authors:** Günnur Onak, Ozan Karaman

**Affiliations:** 1 Tissue Engineering and Regenerative Medicine Laboratory, Department of Biomedical Engineering, İzmir Katip Çelebi University, İzmir, Turkey; 2 Bonegraft Biomaterials Co., Ege University Technopolis, Bornova, İzmir, Turkey

**Keywords:** nanofibers, biomineralization, non-thermal atmospheric plasma, bone tissue engineering

## Abstract

Surface modification by non-thermal atmospheric plasma (NTAP) treatment can produce significantly higher carboxylic groups on the nanofibers (NF) surface, which potentially can increase biomineralization of NF via promoting glutamic acid (GLU) templated peptide conjugation. Herein, electrospun poly(lactide-co-glycolide) (PLGA) scaffolds were treated with NTAP and conjugated with GLU peptide followed by incubation in simulated body fluids for mineralization. The effect of NTAP treatment and GLU peptide conjugation on mineralization, surface wettability and roughness were investigated. The results showed that NTAP treatment significantly increased GLU peptide conjugation which consequently enhanced mineralization and mechanical properties of NTAP treated and peptide conjugated NF (GLU-pNF) compared to neat PLGA NF, NTAP treated NF (pNF) and GLU peptide conjugated NF (GLU-NF). The effect of surface modification on human bone marrow derived mesenchymal stem cells adhesion, proliferation and morphology was evaluated by cell proliferation assay and fluorescent microscopy. Results demonstrated that cellular adhesion and proliferation were significantly higher on GLU-pNF compared to NF, pNF and GLU-NF. In summary, NTAP treatment could be a promising modification technique to induce biomimetic peptide conjugation and biomineralization for bone tissue engineering applications.

## Introduction

Scaffolds composed of hydroxyapatite (HA) and natural or synthetic polymers with tunable mechanical, chemical and physical properties, which can successfully mimic mineralized collagen nanofibers (NF) in bone tissue, are one of the major targets in bone tissue engineering [1, 2]. To fabricate such biomimetic scaffold, many fabrication techniques have been utilized including electrospinning, solvent casting, freeze-drying, laser sintering and 3D printing [3–5]. Among those techniques, electrospinning has been widely used to develop bone extracellular matrix (ECM) mimetic nanofibrous scaffolds by using natural or synthetic polymers [1, 6, 7]. Nanofibrous scaffolds not only mimic the structure of bone ECM, but also provide sufficient surface area for cell adhesion, proliferation and differentiation [8, 9]. However, one of the major drawbacks of using electrospun nanofibrous scaffold on bone tissue engineering is their inadequate mechanical properties [10, 11]. To overcome this limitation and also better imitate the mineralized bone tissue, composite scaffolds have been developed by combining HA, which is the major inorganic element of natural bone, with electrospun scaffolds by generally using blending electrospinning and biomineralizing in simulated body fluid (SBF) [12, 13]. In blending electrospinning technique, HA crystals can only bind physically on the surface of the NF and could be dissociated right after fabrication of the scaffolds. It was previously reported that there was no significant difference in mechanical properties of the electrospun scaffolds developed by blending electrospinning [12, 14]. However, in biomineralization technique, composite nanofibrous scaffolds showed better biocompatibility and significantly higher mechanical properties of scaffolds due to the growth of nano-HA crystals that strongly attached to the surface of the NF [13, 15]. To initiate nano-HA growth on the surface of NF, biological cues that initiate the first step of the biomineralization known as the calcium chelating process should be present on the NF surface [8, 16].

Although natural polymers have biological cues that highly support scaffold mineralization as well as cell adhesion and proliferation [17], they are known to have some disadvantages such as less control over their mechanical properties and fast biodegradability [18, 19]. To address this issue some unmodified synthetic polymers have been used as tissue engineering scaffolding material, but the absence of biological recognition cues on their surface limits their efficient use [20, 21]. Therefore, surface of the synthetic polymers need to be modified to provide functional groups that initiates binding of biological cues that are known to induce biomineralization [22].

There are several surface modification methods to associate biological function into synthetic NF scaffolds [23]. Typically, these methods are based on chemical integration of cell directive peptides or growth factors into the scaffold [24]. The mechanical properties of the polymer should retain while biochemical signals ensures the activity following chemical modification [24]. Surface modification of the scaffolds by using bioactive molecules such as ECM proteins or peptides can induce the desired signaling pathways in cells. The diversity of functional groups on ECM proteins makes peptides a great candidate for tissue engineering applications. In natural bone tissue, nucleation and growth of HA crystals on collagen NF are mediated via bone sialoprotein (BSP) and osteocalcin which are constituent of bone ECM. These non-collagenous protein structure involve glutamic acid (GLU) sequences ranging from 2 to 10 residues mediate nucleation and growth of HA [25]. In our previous study, we conjugated 2-mer GLU peptide on poly(lactide-co-glycolide) (PLGA) NF and investigated the effect of HA deposition to aligned NF on osteogenic differentiation of rat marrow stromal cells (MSCs). In that study, to conjugate biomimetic peptide sequence to accelerate biomineralization, we synthesized low molecular weight acrylated poly (L-lactide) (PLAA), conjugate 2-mer GLU peptide to acrylate groups of PLAA and electrospun high molecular weight PLGA blended low molecular weight PLAA-2-mer GLU compound. The results demonstrated that 2-mer GLU peptide on the surface of PLGA NF enhanced nucleation and growth of HA crystals and accelerated osteogenic differentiation of MSCs and mineralization [15]. In another study, Barati *et al.* added organic acids into modified SBF (mSBF) and investigated biomineralization content on GLU peptide modified polylactic acid (PLA) based NF. It was reported that mineralized CaP content on NF was significantly increased by addition of organic acids to mSBF [26].

Synthetic polymers can be chemically modified with high reactive functional groups, such as carboxyl groups, for direct conjugation of bioactive peptide molecules [27]. Different polymer modification techniques such as pulsed laser deposition, ion beam deposition, covalent immobilization, photo chemical modification and oxygen plasma treatment have been carried out to enhance hydrophilicity and introduce functional groups on the polymeric scaffold surface to act as biological cues [28–30]. Several plasma-based technologies have provided an alternative approach to create thin porous coatings in the nanometer-thickness range that could promote cell attachment and proliferation [31, 32]. Non-thermal atmospheric plasma (NTAP) produces oxygen containing functional groups on the polymer surface, mainly hydroxyl and carboxyl groups by reacting with polymers without altering mechanical properties [33]. Remarkably, when oxygen inserted into the polymer matrix with NTAP treatment, the wettability of polymer surface dramatically increases whereas no surface topography change is observed [34]. The introduction of carboxyl functional groups on the polymer surface could be useful for further conjugation with bioactive peptide for functionalization [35]. Therefore, we considered that it could be used as pre-surface modification technique to effectively immobilize BSP mimetic GLU templated peptide onto the NF.

In this study, we used NTAP treatment as a surface functionalization methodology for effectively conjugating GLU peptide directly to PLGA nanofiber instead of blending electrospinning which are then mineralized in SBF to mimic the biomineralization process and structure of natural bone. To the best of our knowledge this study comprehensively investigated for the first time in the literature the following: (i) determine the effect of NTAP treatment on GLU peptide conjugation and biomineralizing in SBF to form composite scaffolds; (ii) characterization of the morphology, structure and mechanical properties of these surface modified and mineralized scaffolds; and (iii) observing MSCs morphology and proliferation capability on the composite scaffolds.

## Materials and methods

### Peptide synthesis

All the chemicals used for peptide synthesis were purchased from AAPPTEC (Louisville, KY, USA). EEEEEE (Glu-Glu-Glu-Glu-Glu-Glu) peptide were synthesized on 4-methylbenzhydrylamine (MBHA) resin (0.67mmol/g loading capacity) via Fmoc-based solid phase peptide synthesis manually as previously described [15, 32]. Briefly, amino acid coupling based on loading capacity of resin were done with the Fmoc-protected amino acid (2 equiv), hydroxybenzotriazole (HOBt, 2 equiv) and N, *N*-diisopropylethylamine (HBTU, 4 equiv) in Dimethylformamide (DMF) for 3 h. After each coupling reaction, remaining free amine groups were removed by adding to the resin with 10% acetic anhydride/DMF solution for 30 min. Fmoc removal was performed by using 20% piperidine in DMF for 30 min. Each coupling and de-protection reaction were controlled by ninhydrin test. For fluorescein isothiocyanate (FITC) conjugation, Lysin (–Lys) were coupled to EEEEEE sequence to enhance reaction of amino groups to isothiocyanates groups of FITC. After removing Fmoc protecting group of—Lys from EEEEEEK peptide (GLUK), the resins were treated with 5% (v/v) N, *N*-diisopropylethylamine (DIEA) in dichloromethane (DCM). The FITC coupling solution containing 389.4 mg (FITC; Sigma-Aldrich, St. Louis, MO, USA) and 256.8 μl DIEA in 3.0 ml DMF added on the reaction vessel. The peptide was cleaved from resin by using trifluoroacetic acid (TFA): triisopropylsilane (TIPS): water (H_2_O) solution at ratio of 95 : 2.5 : 2.5. DCM and TFA were removed by using the rotary evaporator. The peptide was triturated with ice-cold diethyl cold ether three times and freeze-dried. The peptide was then characterized and purified by Liquid Chromatography Mass Spectrometry (LC-MS) with preparative high-pressure liquid chromatography (HPLC, Agilent 1260 Quaternary LC) equipped with mass spectrometry (Agilent 6530 Q-TOF) with an electrospray ionization (ESI) source as previously described [32].

### Fabrication of nanofibers

The 50 : 50 PLGA (Mw ∼ 90 kDa; Purasorb PDLG5010, Corbion Purac Biomaterials, Gorinchem, The Netherlands) was used for electrospinning as described previously [15]. In brief, a blend of 7 wt% PLGA was dissolved in 1, 1, 1, 3, 3, 3-hexafluoro-2-propanol (HFIP; Matrix Scientific; Columbia) solvent. The polymer solution was transferred and injected from a 5-ml syringe equipped with a 21-gauge needle by using a syringe pump. Aluminum foil was wrapped around the drum and 12 mm circular glass coverslips (VWR, Bristol, CT, USA) were attached to the rotating wheel to collect aligned NF. The aligned NF were collected by an aluminum rotating wheel with previously optimized electrospinning conditions such as a syringe flow rate of 1.0 ml/h, a 20 kV electrical potential, a throw distance of 15 cm and rotation speed of 1200 rpm [15].

### Peptide conjugation of nanofibers

Surface modification of the scaffolds was performed using 1-ethyl-3-(3-dimethylaminopropyl)-carbodiimide and *N*-hydroxysuccinimide (EDC/NHS) chemistry after NTAP treatment. Briefly, NTAP was applied on electrospun NF at 31 kV of output voltage and 1.5 kHz frequency for 45 s with a fixed 1 mm of discharge gap. After NTAP treatment, scaffolds were washed with distilled water three times. A solution containing EDC (2 mM) and NHS (5 mM) in 0.1 M MES buffer (pH 5.3) was added to NF and kept at 37°C for 40 min. The activated NF were immersed with DI water three times to eliminate the unreacted solution. The NF were then treated with EEEEEE peptide solutions at concentration of 1 mM in sterile PBS, followed by incubation at 4°C for 24 h. The NF are washed with PBS before characterization.

### Calcium phosphate crystals nucleation and growth on NF

All the chemicals used for SBF preparation were purchased from Sigma-Aldrich. NF were incubated in a modified 1.5-fold concentrated SBF (1.5x SBF) for 1, 4 and 7 days. The SBF solution was prepared as previously described [36] by dissolving sodium chloride, potassium chloride, calcium chloride monohydrate (CaCl_2_.H_2_O), magnesium chloride hexahydrate (MgCl_2_.6H_2_O), sodium bicarbonate (NaHCO_3_) and monosodium phosphate (NaH_2_PO_4_) in deionized water to a final pH of 4.2. Next, 60 mM NaHCO_3_ solution was added to adjust pH to 7.4. After incubation, the NF were washed three times with deionized water.

### Characterization of nanofibers

Scanning electron microscope (SEM; Carl Zeiss Microscopy, Germany) was used to characterize fiber diameter of electrospun PLGA NF. The NF were acquired with 3 kV accelerating voltage after coating with gold (QUORUM; Q150 RES; East Sussex; UK) at 20 mA for 60 s. NF were analysed with ImageJ software to determine the average fiber size by using the scale bars in the images obtained from the SEM software.

Peptide conjugation on NF were assessed as previously described [15]. Briefly, NF, NTAP treated NF (pNF), GLU peptide conjugated NF (GLU-NF), and NTAP treated and peptide conjugated NF (GLU-pNF) were treated with 5 mg/ml FITC solution prepared in Dulbecco's phosphate-buffered saline (PBS; Cellgro, Herndon, VA, USA) for 4 h. FITC can chemically couple with only reactive amine side chains of GLUK peptide due to the reaction of primary amine groups of GLUK peptide with carboxyl groups of NF which are exposed via NTAP treatment. After conjugation, all groups were dissolved in dimethylsulfoxide (DMSO; Sigma-Aldrich) and fluorescent intensity was measured with a Synergy™ HTX Multi-Mode Microplate Reader (BioTek, Winooski, VT, USA) at emission and excitation wavelengths of 520 and 495 nm, respectively.

Effect of GLU peptide conjugation and NTAP treatment on hydrophilicity of NF, pNF, GLU-NF and GLU-pNF surface was assessed by contact angle measurements using KSV Attension Theta goniometer (Biolin Scientific, Stockholm, Sweden). Briefly, 4 μl drop of deionized water was applied to the NF, pNF, GLU-NF and GLU-pNF surface, photographed from right and left sides of the droplet over NF surface. The contact angle (θ) was calculated as the mean of all measurements by the system.

The surface topography of NF, pNF, GLU-NF and GLU-pNF were analysed by an Easyscan 2 (Nanosurf AG, Liestal, Switzerland) Atomic Force Microscopy (AFM) with the Nanosurf Easyscan 2 software operating in tapping mode employing a silicon cantilever probe Tap190Al-G (Buged Sensors, Sofia, Bulgaria) with a force constant of 48 N/m and resonance frequency of 190 kHz. Fibers were directly deposited on mica substrate and scanned in dry conditions.

The calcium phosphate (CaP) nucleation amount on the NF was measured using a QuantiChrom calcium assay (Bioassay Systems, Hayward, CA, USA) according to manufacturer's instructions. Briefly, calcium content of the NF was dissolved in 1 M HCl. Next, 50 μl of sample was added to 150 μl of the working solution. Absorbance was measured at a wavelength of 612 nm. Calibration curve was created with reference calcium solutions and measured intensities were correlated to the equivalent amount of Calcium using the calibration curve. The amount of calcium phosphate (CaP) content based on NF mass (wt%) was calculated as described previously [15, 26]. Total CaP mineralized deposit of each sample was determined from the measured calcium content at each time-point (1, 4 and 7 days). In Day 7, the CaP crystal structure on mineralized NF, pNF, GLU-NF and GLU-pNF was determined by a 405S5 wide-angle X-ray diffractometer (XRD; X'Pert Pro, Philips, Eindhoven, The Netherlands) with CuKα radiation source at 30 kV as previously demonstrated [15]. The background spectra of neat PLGA NF were subtracted from each group to better identify CaP peaks. Commercial nano-HA (Sigma-Aldrich) was also analysed with XRD.

The tensile modulus of CaP nucleated NF, pNF, GLU-NF, GLU-pNF (20 × 5 mm) was measured with a computer-controlled Shimadzu Autograph 194 AG-IC Series universal testing machine (Shimadzu Corp., Kanagawa, Japan) with a strain rate of 0.033/s at ambient conditions as previously described [15]. Tensile modulus of each group was calculated from the slope of the linear region of the stress–strain curve.

### Cell attachment and proliferation analysis

Human bone marrow derived mesenchymal stem cells (hMSCs) (HMSC-AD-500, CLS cell lines Service, Lot#102, Eppelheim, Germany) were cultured at 37 C and 5% CO_2_ with Dulbecco’s Modified Eagle’s Medium (DMEM) (Sigma-Aldrich) supplemented with 10% fetal bovine serum (FBS) (Sigma-Aldrich, Steinheim, Germany), 1% L-glutamine (Genaxxon BioScience, Ulm, Germany) and 0.1% penicillin/streptomycin (Genaxxon BioScience). The cells at passage three were used by keeping in exponential phase.

For cell seeding, edges of the NF were attached to 12 mm diameter of circular glass coverslips by a medical-grade silicone sealant (Dow Corning, Midland, MI, USA). The NF were sterilized by ultraviolet (UV) radiation for 1 h and immersed in 70% ethanol for 30 min as described previously [15]. After sterilization, NF were conditioned in basal medium supplemented with 10% FBS, 100 units/ml penicillin, 100 μg/ml streptomycin for 1 h. hMSCs were seeded on the NF at a density of 1 × 10^5^ cells/cm^2^.

Cell proliferation analysis was evaluated with 3- (4, 5-dimethylthiazol-2-yl)-2, 5-diphenyltetrazolium bromide (MTT) (Vybrant^®^ MTT Cell Proliferation Assay Kit, Invitrogen, Waltham, MA, USA) assay according to the manufacturer’s instructions at 1, 4 and 7 days as previously described [32]. Briefly, MTT dye (10% in culture medium) was added to seeded cells and incubated for 4 h at 37°C. Next, the medium was replaced with 500 μl dimethyl sulfoxide (DMSO, Sigma-Aldrich) to dissolve the formazan crystals, and the absorbance was measured at 570 nm using a Synergy™ HTX Multi-Mode Microplate Reader (BioTek).

To observe the hMSCs morphology seeded on NF, pNF, GLU-NF and GLU-pNF, actin filaments and cell nuclei were stained using the actin cytoskeleton/focal adhesion staining kit by phalloidin and 4', 6-Diamidino-2-Phenylindole (DAPI), respectively according to manufacturer’s instructions (Merck Millipore, Billerica, MA, USA) for observation of cell morphology on NF surface as explained elsewhere [32]. Briefly, cell-seeded NF were immersed with PBS twice, fixed with 4% paraformaldehyde (Sigma-Aldrich), permeabilized with 0.1% Triton X-100 in PBS and blocked with 1.5% bovine serum albumin in PBS. NF were then incubated with 0.16 µM phalloidin in PBS for 1 h at 4°C and 300 nM DAPI for 5 min. The stained samples were visualized with a fluorescent microscope.

### Statistical analysis

All data are statistically analysed by two-way ANOVA (SPSS 12.0, SPSS GmbH, Germany) and the Student–Newman–Keuls method as a *post hoc* test. Significant differences between groups were determined at *P*-values at least <0.05. (*P < 0.05, **P < 0.01, ***P < 0.001).

## Results

### Characterization of GLU peptide conjugated nanofibers

**Figure 1:**
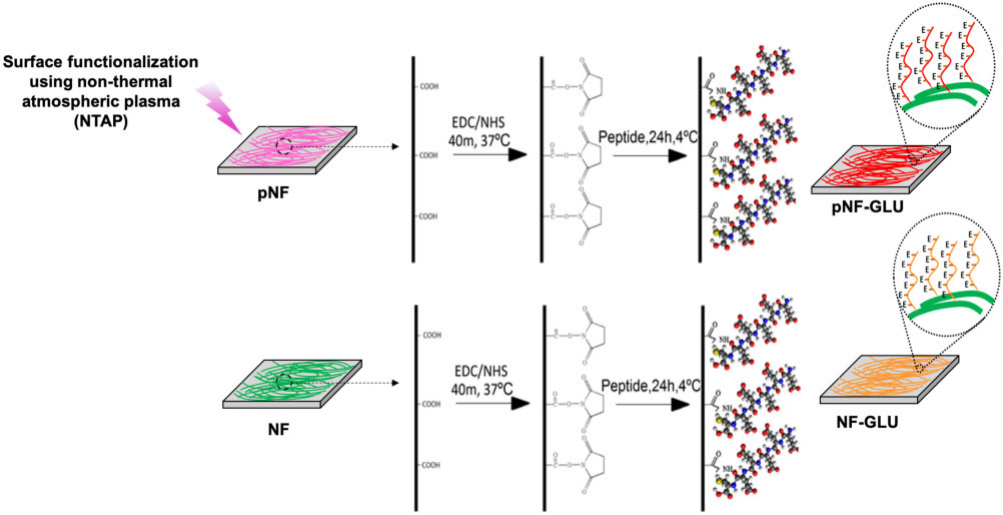
Schematic diagram of NTAP assisted conjugation of glutamic (E) acid templated peptides on PLGA nanofibers with EDC/NHS chemistry. Experimental groups are neat NF, NTAP treated NF (pNF), GLU peptide conjugated NF (without NTAP treatment) (GLU-NF) and NTAP treated GLU peptide conjugated NF (GLU-pNF)

**Figure 2:**
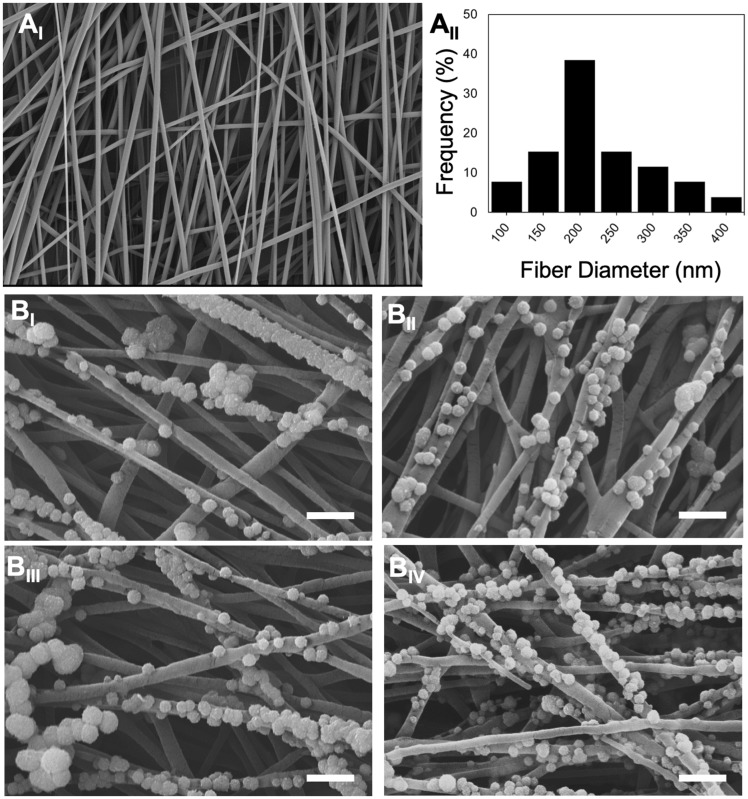
Scanning electron microscopy images of PLGA nanofibers (NF) (A_I_) (scale bar represents 1 µm), histogram showing nanofiber diameters distribution (A_II_), mineralized neat NF (NF, B_I_), mineralized NTAP treated NF (pNF, B_II_), mineralized GLU peptide conjugated NF (without NTAP treatment) (GLU-NF, B_III_), mineralized NTAP treated GLU peptide conjugated NF (GLU-pNF, B_IV_) (scale bar represents 1 µm)

GLU peptide conjugation on to the surface of NTAP treated and non-treated NF were schematically shown in Fig. 1. A SEM photomicrograph of PGLA electrospun NF is given in Fig. 2A_I_. NF diameter distribution histogram (Fig. 2A_II_) was calculated by using ImageJ. Diameter of NF used throughout the study ranged from 76 to 374 nm and the mean diameter was calculated as 266 ± 14 nm. Additionally, the images of the NF, pNF, GLU-NF and GLU-pNF after incubation in 1.5x SBF for 7 days are shown in Fig. 2B_I_, B_II_ and B_III_, respectively. Although CaP crystals were observed in both groups, maximum CaP deposition was achieved in GLU-pNF.

**Figure 3:**
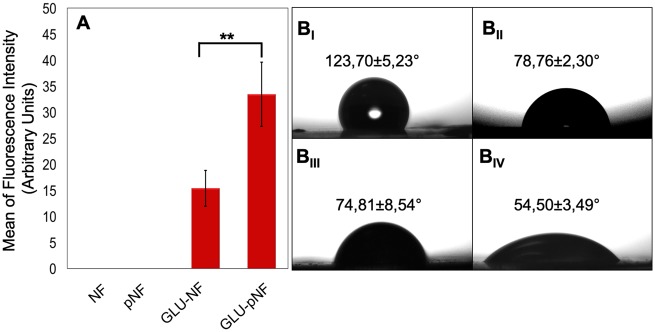
Mean of fluorescence intensity of neat NF, NTAP treated NF (pNF), GLU peptide conjugated NF (without NTAP treatment) (GLU-NF) and NTAP treated GLU peptide conjugated NF (GLU-pNF) after dissolving in DMSO (A_I_), measurement of contact angle of NF (B_I_), pNF (B_II_), GLU-NF (B_III_) and GLU-pNF (B_IV_)

The peptide conjugation efficacy on NF surface was compared by measuring FITC intensity of dissolved FITC labeled GLU peptide. Briefly, FITC labeled GLU peptide were conjugated on the electrospun NF and NTAP treated NF to show the amount of peptide coverage. Then, fibers were dissolved, and FITC intensities of NF, pNF, GLU-NF and GLU-pNF were measured. The mean fluorescence intensity of each sample was also measured by using three different samples. As shown in Fig. 3A, the maximum fluorescence intensity was observed with GLU-pNF, whereas no fluorescence was observed in negative controls (NF and pNF).

Water contact angle (θ) measurement was evaluated to indicate the impact of peptide conjugation on NTAP treated and non-treated PLGA NF on hydrophilicity by dripping 10 µl of deionized water to the surface. The contact angle measurements were conducted on NF, pNF, GLU-NF and GLU-pNF. Results demonstrated that after NTAP treatment the water contact angle of NF dropped from 123.70 ± 5.73° to 71.78 ± 2.41° (Fig. 3B). Furthermore, after conjugation of GLU peptide the water contact angle (θ) of non-NTAP treated NF decreased from 123.70 ± 5.73° to 74.81 ± 8.54°, while NTAP treated NF dropped from 71.78 ± 2.41° to 54.50° ± 3.49°.

**Figure 4:**
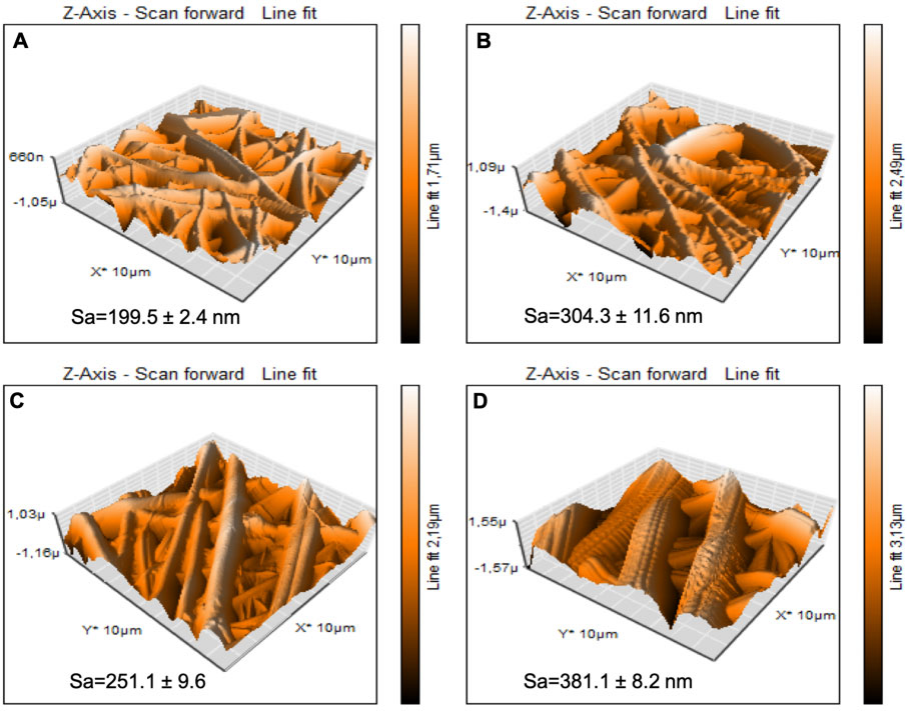
Representative 3D topographical view of neat NF (A), NTAP treated NF (pNF, B), GLU peptide conjugated NF (without NTAP treatment) (GLU-NF, C) and NTAP treated GLU peptide conjugated NF (GLU-pNF, D) obtained by AFM

**Figure 5:**
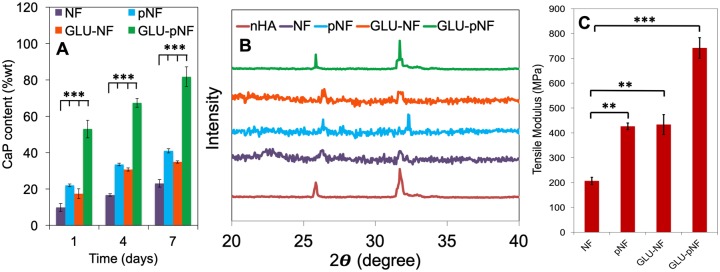
Calcium content of calcium phosphate (CaP) deposited on neat NF, NTAP treated NF (pNF), GLU peptide conjugated NF (without NTAP treatment) (GLU-NF) and NTAP treated GLU peptide conjugated NF (GLU-pNF) after 1, 3 and 7 days incubation in modified 1.5xSBF (A) and comparison of X-ray diffraction spectra of CaP deposited on NF, pNF, GLU-NF and GLU-pNF after 7 days incubation in modified 1.5xSBF (B). Tensile modulus of neat NF, NTAP treated NF (pNF), GLU peptide conjugated NF (without NTAP treatment) (GLU-NF) and NTAP treated GLU peptide conjugated NF (GLU-pNF) (C)

Roughness analysis of NF, pNF, GLU-NF and GLU-pNF was conducted by using AFM. The areal average surface roughness (Sa) of the NF was 199.5 ± 2.4 nm (Fig. 4A). After NTAP treatment, the surface roughness dramatically increased to 304.3 ± 11.6 nm (Fig. 4B). Additionally, GLU peptide conjugation on NF showed a slight increase in roughness (251.1 ± 9.6) (Fig. 4C). Similarly, GLU peptide conjugation after NTAP treatment increased the Sa value from 304.3 ± 11.6 nm to 381.1 ± 8.2 nm (Fig. 5D).

### Biomineralization of GLU peptide conjugated nanofibers

Calcium content deposited on NF, pNF, GLU-NF and GLU-pNF after incubation in 1.5x SBF for 1, 4 and 7 days is shown in Fig. 5A. CaP content wt% on both groups constantly increased as incubation time changed from 1 to 4 and 7 days and similar patterns were observed on each time point. In detail, calcium content on GLU-pNF at Day 1 (53.1 ± 4.8 wt%) was significantly high compared to NF (9.9 ± 2.1 wt%; *P* < 0.001), GLU-NF (17.5 ± 4.7 wt%; *P* < 0.001) and pNF (22.1 ± 1.1 wt%; *P* < 0.001), respectively. A similar pattern was observed at Day 4 where GLU-pNF (67.3 ± 2.1 wt%) had significantly high calcium content compared to NF (16.8 ± 1.7 wt%; *P* < 0.001), GLU-NF (30.7 ± 2.1 wt%; *P* < 0.001) and pNF (33.5 ± 2.8 wt%; *P* < 0.001), respectively. At Day 7, the calcium content on pNF (41.7 ± 1.6 wt%; *P* < 0.001), GLU-NF (34.9 ± 2.3 wt%; *P* < 0.001) and GLU-pNF (81.7 ± 5.1 wt%; *P* < 0.001) significantly higher than NF (23.2 ± 2.1 wt%). It was also observed that GLU-pNF had significantly high content of calcium at Day 7 compared to NF (*P* < 0.001), pNF (*P* < 0.001) and GLU-NF (*P* < 0.001).

The XRD spectra of commercial HA (red), NF (purple), pNF (blue), GLU-NF (orange) and GLU-pNF (green) after 7 days of incubation in 1.5x SBF are shown in Fig. 5B. Results showed that the crystals deposited only on GLU-pNF showed characteristic HA peaks centered at 25.8° and 31.8° [37] while NF, pNF and GLU-NF showed slight peaks that did not match specific HA peaks.

Mechanical characterization of CaP deposited NF, pNF, GLU-NF and GLU-pNF was conducted and tensile modulus of each group is presented in Fig. 5C. The tensile modulus of CaP deposited NF, pNF, GLU-NF and GLU-pNF were measured as 206 ± 14 MPa, 426 ± 23 MPa, 433 ± 69 MPa and 742 ± 41 MPa, respectively.

### Cell proliferation and morphology on GLU peptide conjugated nanofibers

**Figure 6:**
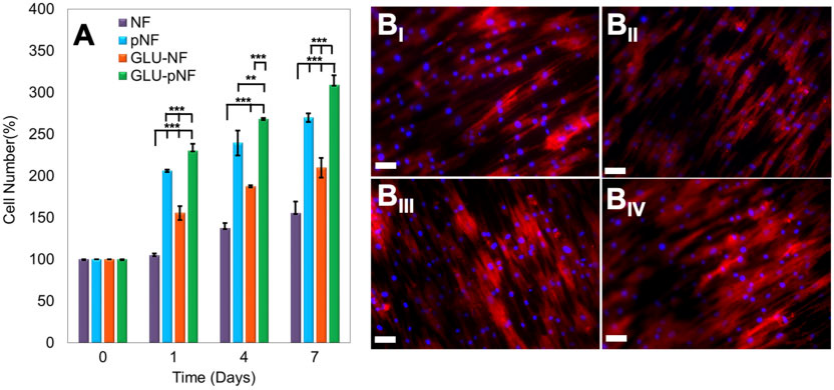
The Percentage of increase in cell number at 1, 3 and 7 days after seeding cells on neat NF, NTAP treated NF (pNF), GLU peptide conjugated NF (without NTAP treatment) (GLU-NF) and NTAP treated GLU peptide conjugated NF (GLU-pNF). (A_I_) Morphology of human marrow stromal cells (hMSCs) seeded on NF (B_I_), pNF (B_II_), GLU-NF (B_III_) and GLU-pNF (B_IV_). PLGA NF incubated in basal media for 7 days. In the images, cell nuclei and cytoskeletal actin are stained with DAPI (blue) and phalloidin (red) (scale bar represents 50µm)

Cell proliferation on CaP nucleated NF, pNF, GLU-NF and GLU-pNF was evaluated by MTT assay with respect to incubation time of 1, 4 and 7 days (Fig. 6A). At Day 1, the ratio of cell number increase on NF, GLU-NF, pNF and GLU-pNF were 5 ± 3%, 55 ± 8%, 106 ± 2% and 130 ± 4%, respectively. After Day 4, the percentage of cell number increase was the highest on the GLU-pNF (168 ± 11%), followed by pNF (139 ± 14%), GLU-NF (87 ± 2%) and NF (37 ± 6%). Moreover, at Day 7, cell number increase on GLU-pNF (209 ± 3%; *P* < 0.001) were significantly higher than pNF (170 ± 5), GLU-NF (110 ± 11) and NF (55 ± 14).

The morphology of the hMSCs on CaP nucleated NF, pNF, GLU-NF and GLU-pNF was examined after culturing in basal medium for 7 days using a fluorescence microscopy (Fig. 6B_I-IV_). Fluorescence microscope images showed that hMSCs effectively attached and spread on each group. Although the number of cells changed within groups and with respect to culture time, there was no difference observed in hMSCs morphology when seeded on CaP deposited NF, pNF, GLU-NF and GLU-pNF.

## Discussion

There is a growing demand for biomimetic synthetic scaffolds with improved biological properties for accelerated bone regeneration due to the requirement of enhanced differentiation as well as cell adhesion, proliferation[38, 39]. Composite nanofibrous scaffolds have been extensively used for bone tissue engineering applications and named as one of the most ideal scaffolds since it can mimic the structure of bone and presents extensive surface area for cell adhesion [40, 41]. One of the important drawbacks defined for synthetic nanofibrous scaffolds is the difficulty of CaP nucleation exactly on the structure of NF which directly affects mechanical properties and biological response [42]. In the present study, we modified the PLGA NF surface with NTAP treatment to increase GLU peptide conjugation and characterize the influence of such modification on biomineralization as well as hMSCs adhesion, proliferation and morphology.

Micrographs of NF, pNF, GLU-NF and GLU-pNF after biomineralization are shown in Fig. 2B_I-III_. The results demonstrated that CaP crystals were in the average size of 110 nm ± 12 and increased by conjugation of GLU peptide on the NF. It was also observed that NTAP treatment caused higher deposition of CaP on NF. Our results were consistent with Kim *et al.* where they reported increased mineralization of CaP crystals via NTAP treatment on poly(∈-caprolactone) (PCL) scaffolds [43]. GLU-pNF group showed high content of CaP crystals compared to NF, pNF and GLU-NF group. Moreover, it could be observed from the SEM micrographs that GLU peptides act as nano-CaP crystals nucleation points as in biomineralization.

Fluorescence intensity of GLU-pNF group showed significantly high fluorescence intensity compared to GLU-NF due to facilitated peptide conjugation (Fig. 3A). It is speculated that increased carboxyl groups on the NF surface following NTAP treatment resulted in higher peptide conjugation. These results are also consistent with contact angle measurements. Contact angle results indicated that surface hydrophilicity increased by NTAP treatment and negatively charged GLU peptide conjugation. As shown in Fig. 3B_I-IV_, the contact angle (θ) dropped from 123.70 ± 5.23° to 54.50 ± 3.49° in NTAP treated NF, whereas the contact angle (θ) following GLU peptide conjugation dropped from 123.70 ± 5.23° to 74.81 ± 8.54°. It was considered that lower contact angle could directly be related to higher GLU peptide conjugation with the help of NTAP treatment. These results confirmed by Rezaei *et al.* who observed that oxygen DBD plasma treatment resulted in the breakdown of C–C and C–H bonds on the PMMA surface leading to introduction of carbon radicals and production of functional oxygen-containing groups such as COOH and O–H on the PMMA surface [44]. It is important to maximize introduction of—COOH groups to polymer surface to enhance peptide conjugation. For that purpose, NTAP treatment increased peptide conjugation by producing functional oxygen-containing groups such as COOH and OH. Hence, effective peptide conjugation might be resulted in increased hydrophilicity in addition to NTAP related functional oxygen groups on the surface. Similar to our results, Yang *et al.* [45] and Karaman *et al.* [15] demonstrated that peptide immobilized NF became more hydrophilic. Therefore, with the support of these results, it is speculated that NTAP assisted GLU peptide conjugation significantly increased GLU peptide content on NF leading to increased CaP content.

Surface topographies of NF, pNF, GLU-NF and GLU-pNF were demonstrated in Fig. 4A–D, respectively. According to the results, NTAP treatment and GLU peptide conjugation increased the surface roughness (Sa) of NF. In addition, the surface roughness increase is higher on pNF to GLU-pNF than NF to GLU-NF. It might be considered that NTAP treatment resulted with efficient peptide conjugation on NF surface. In a recent study, Man *et al.* fabricated peptide conjugated electrospun fibers using a polyvinyl pyrrolidone/bovine serum albumin/rhTGF-β1 composite solution and poly(ε-caprolactone) (PCL) to develop a co-delivery system of rhTGF-β1 and MSCs affinity peptide. They reported that peptide conjugation on scaffolds resulted in increased roughness [46, 47]. In another study, Deng *et al.* prepared peptide-decorated PCL nanofibrous microenvironment with electrospinning technique and conjugated with vitronectin peptide to enhance the osteogenic potential of human pluripotent stem cells (hPSCs) *in vitro*. Their results demonstrated that addition of peptide also increased the surface roughness of the modified PCL substrates [46, 47].

Calcium assay results suggested that GLU-pNF showed significantly higher CaP deposition than NF, pNF and GLU-NF (Fig. 5A). It was speculated that enhanced negatively charged functional groups produced after NTAP treatment following with GLU conjugation caused a sharply increase in calcium content. In our previous study, PLAA-GLU peptide conjugate was blended with high MW PLGA and electrospun fibers were fabricated. CaP content ratio based on NF mass results indicated that on single layer GLU-NF nanofibers maximum 49.2 ± 2.1% CaP content was obtained [15]. However, with NTAP assisted conjugation we conducted in this study, significantly higher CaP content (81.7 ± 5.1%) was determined. These results confirmed that effective peptide conjugation with initial NTAP treatment significantly increased the amount of CaP content mineralized on the surface of the NF.

NTAP treatment of synthetic NF exposes a variety of functional groups on them, including amino, carboxyl and hydroxyl groups [48]. Cui *et al.* reported that among developed functional amino, carboxyl and hydroxyl groups on Poly(DL-lactide) (PDLLA) fibers, carboxyl groups have more potent influence on HA nucleation and growth in SBF due to the strong electrostatic attraction of carboxyl groups and calcium ions [49]. Our results are consistent with Sarvestani *et al.*, where they investigated the effect of 6-mer glutamic acid peptide (Glu6) conjugated to synthetic hydrogel to improve mechanical properties by enhancing interaction with CaP nanocrystals. The results indicated that conjugation of Glu6 peptide increased carboxylic groups on the hydrogels due to the available carboxyl group on each GLU and higher interaction of Glu6 peptide with CaP crystals enhanced shear modulus of composite hydrogel [50]. All these studies and our results suggest that GLU peptide conjugation after NTAP treatment causes formation of significantly higher number of carboxyl groups on NF compared to NF, pNF and GLU-NF groups, which enhances interaction with calcium ions in SBF and CaP mineralization.

The CaP crystal characteristics were determined via XRD analysis. The results demonstrated that only GLU-pNF group resembled HA characteristic CaP crystals on the NF (Fig. 5B). Based on these findings we considered that enhanced GLU peptide conjugation not only increased the content of CaP crystals but also guide the calcium ions adhesion and further reaction to transform into HA crystals known to be the stoichiometrically stable phase of CaP and major content of bone inorganic phase. Although GLU-NF and GLU-pNF groups theoretically directed mineralization through conjugated GLU peptide, limited GLU peptide conjugation was observed on GLU-NF group due to the lack of available carboxyl groups (Fig. 3A). Therefore, CaP crystals characteristics were influenced and shifted HA specific XRD spectrum to brushite [51]. As already stated, the substrate surface adsorbs calcium ions and initial nucleation sites are aggregated near the surface during mineralization [52]. It is speculated that limited carboxyl groups due to less GLU peptide conjugation inhibits formation of HA which is stoichiometrically stable form of CaP crystals [15]. In a previous study, Tavafoghi *et al.* investigated the effect of a negatively charged GLU and positively charged Arginine. It was reported that negatively charged functional groups were more effective than neutral and positive groups in HA precipitation of due to hydrogen bond, carboxyl groups and electrostatic attraction [53].

Tensile modulus of CaP deposited NF, pNF, GLU-NF and GLU-pNF are presented in Fig. 5C. Results showed that increased nano-CaP deposition on GLU-pNF significantly enhanced tensile modulus compared to NF, pNF and GLU-NF. It was also indicated that CaP deposited pNF and GLU-NF significantly higher tensile modulus level than NF. One possible explanation for such trend is the increase and homogenous deposition of nano-CaP influence mechanical properties of NF [54, 55]. Compared to direct addition of CaP into electrospinning solution to develop CaP deposited NF scaffolds, nucleating the nano-CaP on NF was reported as more effective technique due to cause of immediate precipitation of nano-CaP before ejection [56, 57]. For instance, a negatively charged surface enhances the nucleation of CaP, leading to a more uniform, thick, dense coating and subsequently improved mechanical properties which are considered important for bone tissue engineering applications [58].

Proliferation of hMSCs on CaP nucleated NF, pNF, GLU-NF and GLU-pNF was presented in Fig. 6A. Faster proliferation of hMSCs was observed with increasing the CaP content on NF. It could be related to higher CaP content by modifying the surface with NTAP treatment and GLU peptide conjugation following with mineralization formed better biomimetic surface structure of bone ECM. It was previously noted that surface properties of scaffolds including wettability, roughness and chemical functionalities with bioactive molecules directly affect cell attachment and proliferation [59]. Our results were consistent with those of Birhanu *et al.* where they applied NTAP treatment following CaP deposition on electrospun composite PLLA/P123 NF and demonstrated that increased hydrophobicity and roughness positively influence cellular adhesion and proliferation [60]. Rough surfaces provide higher surface area which in turn increases the interaction between NF surface and integrin binding points of the cells [61]. Although different proliferation profiles were observed on NF, pNF, GLU-NF and GLU-pNF, there was no significant difference on morphology of hMSCs suggesting that hMSCs kept their stemness ability and most likely did not start differentiation when cultured in basal media.

Promoting biomineralization via effective conjugation of biomimetic peptides on synthetic NF could potentially be a strong alternative to scaffolds for guided bone regeneration in tibial and mandibular defects [62]. Mesenchymal stem cells seeded mineralized electrospun scaffolds would be acting as a barrier to prevent invasion of soft tissue toward bone defect at the same time accelerate bone regeneration due to biocomposite structure of the scaffold.

## Conclusion

In this study, the effect of NTAP treatment mainly on GLU peptide conjugation, mineralization of NF, mechanical properties and hMSC proliferation was investigated. NTAP treated and non-treated PLGA NF were conjugated with GLU peptide and mineralized in the SBF. The results revealed that NTAP treatment significantly increased GLU peptide conjugation which further enhanced CaP nucleation on NF. Mechanical properties of NF improved by increasing the deposition of nano-CaP on NF. In addition, increasing the CaP content on NF NTAP treatment and GLU peptide conjugation enhanced proliferation of hMSCs. All these results together implied that NTAP treatment following with biomimetic GLU peptide conjugation could potentially be a promising technique to develop efficient CaP nucleated NF for bone tissue engineering applications.
